# Multilevel regulation of non‐photochemical quenching and state transitions by chloroplast NADPH‐dependent thioredoxin reductase

**DOI:** 10.1111/ppl.12914

**Published:** 2019-02-08

**Authors:** Lauri Nikkanen, Manuel Guinea Diaz, Jouni Toivola, Arjun Tiwari, Eevi Rintamäki

**Affiliations:** ^1^ Molecular Plant Biology, Department of Biochemistry University of Turku Turku Finland

## Abstract

In natural growth habitats, plants face constant, unpredictable changes in light conditions. To avoid damage to the photosynthetic apparatus on thylakoid membranes in chloroplasts, and to avoid wasteful reactions, it is crucial to maintain a redox balance both within the components of photosynthetic electron transfer chain and between the light reactions and stromal carbon metabolism under fluctuating light conditions. This requires coordinated function of the photoprotective and regulatory mechanisms, such as non‐photochemical quenching (NPQ) and reversible redistribution of excitation energy between photosystem II (PSII) and photosystem I (PSI). In this paper, we show that the NADPH‐dependent chloroplast thioredoxin system (NTRC) is involved in the control of the activation of these mechanisms. In plants with altered NTRC content, the strict correlation between lumenal pH and NPQ is partially lost. We propose that NTRC contributes to downregulation of a slow‐relaxing constituent of NPQ, whose induction is independent of lumenal acidification. Additionally, overexpression of NTRC enhances the ability to adjust the excitation balance between PSII and PSI, and improves the ability to oxidize the electron transfer chain during changes in light conditions. Thiol regulation allows coupling of the electron transfer chain to the stromal redox state during these changes.

**Abbreviations**
AL: actinic light
Ax: antheraxanthin
CEF: cyclic electron flow
ECS: electrochromic shift
EPR: electron paramagnetic resonance
Fd: ferredoxin
GL: growth light
HL: high light
LL: low light
NPQ: non‐photochemical quenching
OE: overexpression
OPPP: oxidative pentose phosphate pathway
PC: plastocyanin
pmf: proton motive force
PQ: plastoquinone
TRX: thioredoxin
TR: thioredoxin reductase
NTRC: NADPH‐dependent thioredoxin reductase
VDE: violaxanthin de‐epoxidase
Vx: violaxanthin
ZE: zeaxanthin epoxidase
Zx: zeaxanthin

## Introduction

THIOREDOXINS (TRXs) are protein oxidoreductases that control the structure and function of proteins by cleavage of a disulphide bond between the side chains of two cysteine residues. Oxidized TRXs are reactivated by THIOREDOXIN REDUCTASES (TR) and a TR‐dependent reduction of TRXs is called a TRX system. Chloroplasts contain two TRX systems with distinct reductants. In the ferredoxin‐TRX system (Fd‐TRX) reducing equivalents are mediated via photosynthetically reduced ferredoxin (Fd) to FERREDOXIN‐THIOREDOXIN REDUCTASE, which subsequently activates at least *f*‐, *m*‐, *y*‐ and *x*‐type TRXs (Schürmann and Buchanan 2008, Yoshida and Hisabori 2017). A single enzyme, the NADPH‐DEPENDENT THIOREDOXIN REDUCTASE (NTRC), has both a TR and a TRX domain and constitutes the other plastidial TRX system (Serrato et al. 2004). NTRC maintains activity in dark and low light conditions (Nikkanen et al. 2018) as its reductant, NADPH, is produced by the oxidative pentose phosphate pathway (OPPP) also in the absence of illumination. NTRC has been demonstrated to be a crucial regulator of photosynthesis, chloroplast metabolism and reactive oxygen species (ROS) detoxification (Pérez‐Ruiz et al. 2006, Michalska et al. 2009, Richter et al. 2013, Carrillo et al. 2016, Naranjo et al. 2016, Nikkanen et al. 2016). Crosstalk and partial redundancy between the two TRX systems exist in the control of photosynthesis and ROS metabolism (Toivola et al. 2013, Thormählen et al. 2015, Nikkanen et al. 2016, Pérez‐Ruiz et al. 2017), but both systems are essential for normal growth and development of plants (Serrato et al. 2004, Wang et al. 2014).

It has recently been proposed that chloroplast TRXs are also major regulators of photosynthetic redox poise during fluctuations in light intensity (Nikkanen and Rintamäki 2014, Thormählen et al. 2017, Nikkanen et al. 2018). TRXs control the redox homeostasis between the photosynthetic electron transfer chain in thylakoid membranes (PETC) and stromal processes by reversibly activating and deactivating regulatory and photoprotective mechanisms that prevent excessive reduction of the PETC and damage to the photosynthetic apparatus (Rintamäki et al. 2000, Courteille et al. 2013, Strand et al. 2016, Da et al. 2018). One of these mechanisms is non‐photochemical quenching (NPQ), which dissipates excitation energy absorbed by the light‐harvesting antenna of photosystem II (PSII) as heat (Niyogi and Truong 2013). Induction of the major, energy‐dependent component of NPQ (qE) requires acidification of the thylakoid lumen via translocation of protons from stroma to lumen by linear and cyclic electron flow (CEF), which then results in downregulation of thylakoid electron flow (Demmig‐Adams et al. 2012, Armbruster et al. 2017). Low lumenal pH causes protonation of the photosystem II Subunit S (PsbS; Li et al. 2000) and activation of the lumenal xanthophyll cycle enzyme VIOLAXANTHIN DE‐EPOXIDASE (VDE), resulting in accumulation of zeaxanthin (Zx, Jahns and Holzwarth 2012). Both PsbS protonation and Zx formation promote the induction of qE (Sylak‐Glassman et al. 2014). On the stromal side of the thylakoid membrane, ΔpH‐independent ZEAXANTHIN EPOXIDASE (ZE) converts Zx back to violaxanthin (Vx) and contributes to relaxation of NPQ (Jahns and Holzwarth 2012). Induction and relaxation kinetics of PsbS‐dependent qE are fast, occurring within one minute of onset or cessation of illumination, respectively (Li et al. 2002). Zx‐dependent quenching (qZ) and photoinhibition of PSII reaction centers under excessive illumination (qI) cause slow‐induced and slow‐relaxing components of NPQ (Tyystjärvi 2013, Ruban 2016, Kress and Jahns 2017). Recently, still another slow‐relaxing component of NPQ denoted qH was identified, which is induced by light or cold stress, is independent of trans‐thylakoid ΔpH and PsbS, requires lumenal lipocalin protein, and is repressed by the SUPPRESSOR OF QUENCHING 1 (SOQ1) protein in the thylakoid membrane that has a lumenal TRX‐like domain (Brooks et al. 2013, Malnoë et al. 2017).

State transitions constitute the qT‐component of NPQ and involve alteration of the sizes of PSII and PSI antenna cross sections through regulation of the reversible association of mobile LHCII proteins with the photosystems (for reviews, see Allen 2003, Ruban and Johnson 2009, Rochaix et al. 2012). This is achieved via dynamic phosphorylation and de‐phosphorylation of LHCB1 and LHCB2 proteins by the STATE TRANSITION 7 (STN7) kinase and THYLAKOID‐ASSOCIATED PHOSPHATASE 38 (TAP38), respectively (Bennett et al. 1980, Bellafiore et al. 2005, Pribil et al. 2010). The STN7 kinase is activated by reduction of the plastoquinone (PQ) pool and binding of PQH_2_ to the cytochrome (Cyt) *b6f* complex, and inactivated in high light (HL) by TRXs (Vener et al. 1997, Rintamäki et al. 2000, Lemeille et al. 2009).

Alteration of stromal thiol redox state has been shown to affect NPQ (Naranjo et al. 2016, Da et al. 2018), CEF (Courteille et al. 2013, Strand et al. 2016, Nikkanen et al. 2018) and reversible phosphorylation of LHCII proteins (Rintamäki et al. 2000). TRXs may affect induction of NPQ via inhibition of VDE by TRX‐mediated reduction of regulatory disulfides (Hall et al. 2010), while thiol regulation of ZE has also recently been demonstrated (Da et al. 2018). Furthermore, we have recently reported that overexpression of NTRC (OE‐NTRC) increases generation of the proton motive force (*pmf*) over the thylakoid membrane (Nikkanen et al. 2018). In OE‐NTRC plants an enhancement of CEF resulted in increased *pmf* in all light conditions, but particularly during dark‐to‐light transitions and sudden increases in light intensity (Nikkanen et al. 2018).

In the current paper, we have examined the molecular background of TRX‐mediated regulation of NPQ and state transitions in plants lacking or overexpressing the *NTRC* gene. Our results suggest that NTRC is required to activate an inhibitory mechanism of NPQ that is independent of formation of trans‐thylakoid ΔpH. Moreover, NTRC overexpression significantly enhances, while NTRC knockout impairs, the plant's ability to redistribute excitation energy between PSII and PSI. In conjunction with other recent reports (Naranjo et al. 2016, Thormählen et al. 2017, Nikkanen et al. 2018), our findings support the hypothesis that NTRC has an important function in adjusting photosynthetic redox poise through either direct or indirect regulation of photoprotective and regulatory mechanisms during changes in light conditions.

## Materials and methods

### Plant material and growth conditions

*Arabidopsis thaliana* wild type (WT) of the Columbia ecotype (Col‐0), T‐DNA knockout mutants of NTRC (At2g41680, SALK_096776, Lepistö et al. 2009) and STN7 (AT1G68830, SALK_073254, Bellafiore et al. 2005) as well as the NTRC overexpression lines (Toivola et al. 2013), were grown in a photoperiod of 8 h light and 16 h darkness at 23 °C under 200 μmol of photons m^−2^ s^−1^ (growth light, GL), using Philips TL‐D 36 W/840a fluorescent tubes as light sources.

### Protein extraction and SDS‐PAGE

Thylakoid membranes and soluble proteins were extracted from leaves as previously reported (Lepistö et al. 2009). Protein content was determined with the Bio‐Rad Protein Assay Kit and chlorophyll (Chl) content according to Porra et al. (1989). Sodium dodecyl sulfate polyacrylamide gel electrophoresis (SDS‐PAGE) and Western blotting were done as described earlier (Nikkanen et al. 2016). PVDF membranes were probed with specific antibodies against PsbS (Agrisera, AS09 533), ZE (Agrisera, AS08 289), VDE (Eskling and Åkerlund 1998), STN7 (Agrisera, AS16 4098), and phosphothreonine (P‐Thr, New England Biolabs). A horseradish peroxidase (HRP)‐conjugated goat anti‐rabbit secondary antibody (Agrisera, AS09 602) was used for detection of proteins. All blot images are representative of at least three biological replicates. Quantifications of protein content were performed using the ImageJ software (Schneider et al. 2012) and normalized according to the intensity of Li‐Cor Revert Total Protein Stain or Coomassie brilliant blue stain. Statistical significance was determined by two‐tailed Student's *t*‐tests for unequal variances. *P* < 0.05 was interpreted as statistically significant.

### Measurement of chlorophyll *a* fluorescence

**Figure 1:**
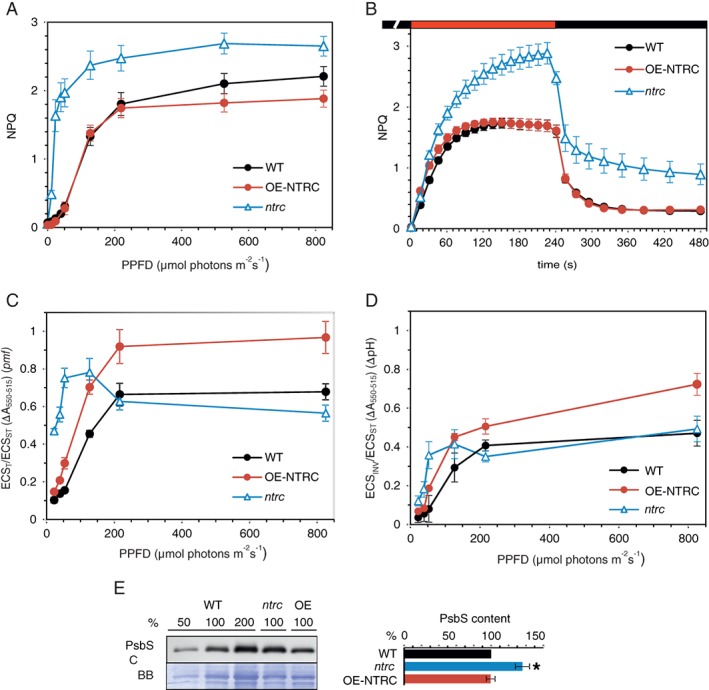
Light response curves of *pmf* and NPQ for WT, OE‐NTRC and *ntrc* leaves. (A) Light response curve of NPQ estimated from Chl *a* fluorescence measurements from WT, OE‐NTRC and *ntrc* leaves. PPFD = photosynthetic photon flux density. Values shown are averages of measurements from eight individual leaves ± SE. (B) Induction of NPQ upon onset of illumination with AL of 166 μmol photons m^−2^ s^−1^ (red bar) and NPQ relaxation after cessation of illumination (black bar) in dark‐adapted WT, OE‐NTRC and *ntrc* leaves. Values shown are averages of measurements from seven to nine individual leaves ±SE. (C) Generation of *pmf* in WT, OE‐NTRC and *ntrc* leaves illuminated at different light intensities. The *pmf* was estimated as the light‐induced change in the ECS signal. Values shown are averages of measurements from three to eight individual leaves ± SE. (D) Contribution of ΔpH to *pmf* in WT, OE‐NTRC and *ntrc* leaves illuminated at different light intensities. Values were estimated as the magnitude of the ECS ‘undershoot’ (ECS_INV_) upon cessation of illumination, as described by Schreiber and Klughammer (2008). Values shown are averages of measurements from three to eight individual leaves ± SE. (E) Content of PsbS in thylakoid membranes isolated from WT, OE‐NTRC and *ntrc*. A representative immunoblot and quantified averages ±SE of three biological replicates are shown. The loading of the gels was based on Chl content of the samples, and Coomassie brilliant blue (CBB) staining is shown as loading control. Statistically significant difference to WT according to Student's *t*‐test (*P* < 0.05) is marked with *.

NPQ was determined from saturating pulse‐induced changes of Chl *a* fluorescence measured with a Dual‐PAM‐100 spectrometer (Walz) and calculated with the Dual‐PAM software (Klughammer and Schreiber 2008) according to Bilger and Björkman (1990). Plants were dark‐adapted for 30 min before all measurements. For the light response curves of NPQ (Fig. 1A), detached leaves were illuminated with actinic light (AL) of 620 nm wavelength. Before the onset of AL and after each 2 min intensity step, a saturating pulse (SP) of 8000 μmol of photons m^−2^ s^−1^ for 800 ms was administered to determine Fm and Fm′. Eight individual leaves were measured from each line. For determination of the induction and relaxation kinetics of NPQ (Fig. 1B), detached leaves were first illuminated with AL of 166 μmol of photons m^−2^ s^−1^ for 4 min. AL was then switched off, and the relaxation of NPQ in darkness was monitored for another 4 min. SPs (8000 μmol of photons m^−2^ s^−1^ and 800 ms) were administered at 15 s intervals during illumination and in darkness at 1, 16, 34, 55, 80, 110, 146, 189 and 240 s after switching off the AL. Seven to nine individual leaves were measured from each line.

Chl *a* fluorescence measurements with a Dual‐PAM‐100 spectrometer were also used to determine the qT, qS and 1/*t*_1/2_ parameters of state transitions (Jensen et al. 2000, Ruban and Johnson 2009). After determining the *F*_0_ and *F*_m_ values from dark‐adapted leaves by an SP of 8000 μmol of photons m^−2^ s^−1^ and 800 ms, we illuminated the leaves with 35 μmol photons m^−2^ s^−1^ of blue actinic light for 20 min to induce state II, after which Fm2 was determined by another SP. Blue actinic light was then supplemented with far red (FR) light (730 nm, 191 μmol photons m^−2^ s^−1^) for another 20 min to induce state I, after which Fm1 was determined by administering a third SP. The *ntrc* mutant suffered from high NPQ even at the low intensity blue AL. This was significantly affecting the Fm2 values and resulting in overestimation of qT in *ntrc*. In order to reliably determine qT, an additional cycle of illumination with blue and blue + FR was applied to sufficiently relieve the qE component of NPQ before determining Fm2 and Fm1 (Fig. S1, Supporting information).

To determine the relative size of PSI antenna cross‐section, Chl *a* fluorescence emission spectra were measured at 77 K with an Ocean Optics QE Pro Spectrometer. Thylakoids were isolated from dark‐adapted leaves or leaves illuminated for 2 h under 40 or 600 μmol of photons m^−2^ s^−1^. Thylakoids were diluted to 5 μg of Chl ml^−1^ in a buffer containing 100 mM sucrose, 50 mM HEPES (pH 7.5), 10 mM NaF, and 10 mM MgCl_2,_ frozen with liquid N_2_ and excited at 440 nm. The raw spectra were normalized at 685 nm for comparison of fluorescence emission bands from PSI.

### Measurement of the electrochromic shift (ECS)

In order to estimate the magnitude of the *pmf*, we measured the light‐induced absorbance difference between 550 and 515 nm referred to as the electrochromic shift (ECS). We used the Dual‐PAM 100 spectrometer and its P515/535 accessory module (Walz; Schreiber and Klughammer 2008), with a measuring light at a 2000 Hz pulse frequency. Plants were dark‐adapted for 30 min, after which detached leaves were illuminated sequentially with 22, 53, and 127, as well as 39, 216, and 826 μmol of photons m^−2^ s^−1^ of AL (620 nm). Each light intensity step was applied for 3 min, after which the AL was switched off for 1 min to monitor the post‐illumination response of the ECS signal. Partitioning of the total *pmf* to ΔpH and ΔΨ was determined from the post‐illumination response of the ECS signal as described by Schreiber and Klughammer (2008). All measured values were normalized with the maximal ECS change (ECS_ST_) induced by a saturating single‐turnover flash of 20 μs and 14 000 μmol photons m^−2^ s^−1^ at the beginning of each measurement from a dark‐adapted leaf. Measurements from three to eight individual leaves were made from each line.

### Measurement of P700, plastocyanin and ferredoxin redox changes

For determination of Fd, plastocyanin (PC) and P700 redox changes, the Dual/Klas‐NIR spectrometer (Walz) was used to record four absorbance differences between 785 and 840, 810 and 870, 870 and 970, as well as 795 and 970 nm, from which the redox changes were deconvoluted as described by Klughammer and Schreiber (2016) and Schreiber (2017). For the measurements from detached leaves of dark‐adapted (30 min) plants shown in Fig. 5, we applied the NIR‐MAX routine (Klughammer and Schreiber 2016) with 3 s of 200 μmol photons m^−2^ s^−1^ actinic light (AL) to determine maximal reduction of Fd, and an FR light of 191 μmol photons m^−2^ s^−1^ for 10 s, followed by a saturating multiple turnover flash (MT, 800 ms, 14 000 μmol photons m^−2^ s^−1^) to obtain maximal oxidation levels of PC and P700. Maximal redox changes where used to normalize the data. For the Fd, PC and P700 redox changes from pre‐illuminated leaves, detached leaves from dark‐adapted plants were first illuminated with red actinic light of 22 μmol photons m^−2^ s^−1^ for 10 min. FR light (191 μmol photons m^−2^ s^−1^) was then turned on for 5 s, followed by a MT (800 ms, 14 000 μmol photons m^−2^ s^−1^). Measurements were made from six (dark‐adapted) or three (pre‐illuminated) individual leaves from each line.

### Determination of functional PSI/PSII ratio by EPR

Electron paramagnetic resonance (EPR) spectroscopy was used to determine the ratio of functional PSI/PSII as described previously (Tiwari et al. 2016). The PSI/PSII ratio in isolated thylakoids was quantified as a ratio of spin numbers obtained by double integration of P700+ signal measured in the presence of DCMU and spin numbers of tyrosine D signal measured as post‐illuminated signal in dark after 5 min of illumination. The spectra were normalized according to Chl *a* concentration, which was determined according to Porra et al. (1989). Full oxidation of P700 was ensured by addition of 10 μM of DCMU to block electron transfer from PSII under an HL intensity (1000 μmol of photons m^−2^ s^−1^) at which P700 signal was already saturated.

### HPLC

To analyze Zx, Ax and Vx content, leaves from 5‐weeks‐old WT, OE‐NTRC and *ntrc* plants were harvested before the onset of light in the morning and after 20 and 40 min of illumination at 200 or 500 μmol photons m^−2^ s^−1^. Plants were then transferred to darkness for 40 min and leaf samples were collected thereafter. Pigments were extracted from leaf discs (5 mm in diameter) using 300 μl of ice‐cold methanol. After centrifugation and filtration of the extracts, photosynthetic pigments were separated by HPLC according to Gilmore and Yamamoto (1991) with a reverse phase C18 column (LiChroCART 125–4, Hewlett Packard), a series 1100 HPLC device with diode array, and a fluorescence detector (Agilent Technologies). Buffer A consisted of acetonitrile–methanol–Tris–HCl buffer 0.1 M pH 8.0 (72:8:3, v/v) and buffer B of methanol–hexane (4:1, v/v). A constant flow rate of 0.5 ml min^−1^ was used. The program started with an isocratic run with buffer A for 4 min followed by a linear gradient for 15 min from 0 to 100% buffer B. The isocratic run of buffer B lasted 26 min. The column was re‐equilibrated between samples for 15 min with buffer A. Pigment standards for Ax, alpha‐carotene, beta‐carotene, Chl *a* and *b*, lutein, 9′‐cis‐neoxanthin, Vx and Zx (DHI Lab Products) were used according to the manufacturer's instructions. Pigment content was calculated as ng mg^−1^ fresh weight.

## Results

### Acidification of the lumen correlates poorly with qE induction in NTRC‐transgenic lines

It has recently been demonstrated that modification of chloroplast thiol‐redox state alters the formation of *pmf* over thylakoid membranes and NPQ induction (Carrillo et al. 2016, Naranjo et al. 2016, Da et al. 2018, Nikkanen et al. 2018). To elucidate the mechanism of the effect of TRXs on NPQ, we measured the induction and relaxation kinetics of NPQ, the magnitudes of both NPQ and *pmf* under different steady‐state light intensities, and determined the relative amounts of proteins and pigments affecting the induction of NPQ in NTRC‐transgenic lines.

Absence of NTRC resulted in increase in NPQ, especially in low light (LL, 40 μmol photons m^−2^ s^−1^) conditions (Fig. 1A). A significant slow‐relaxing component of NPQ also persisted in *ntrc* after cessation of illumination (Fig. 1B). In NTRC‐overexpressing plants NPQ was induced similarly to WT in LL and GL conditions but was slightly lower than WT under HL (Fig. 1A).

As induction of the qE component of NPQ depends on acidification of the thylakoid lumen, we estimated the magnitude of the *pmf* and its ΔpH component from changes in the ECS signal. The lumen was strongly acidified in LL in *ntrc*, whereas in GL and HL similar levels of ΔpH to WT were estimated (Fig. 1C,D). In contrast, NTRC overexpression caused elevation of *pmf* and ΔpH in all light conditions (Fig. 1C,D). However, the steady‐state NPQ remained similar to WT in LL and GL and was lower than WT in HL (Fig. 1A). In LL, the elevated NPQ in *ntrc* can be partly attributed to high ΔpH. However, NPQ induction does not correlate directly with the generation of ΔpH in other conditions or in the OE‐NTRC line.

**Figure 2:**
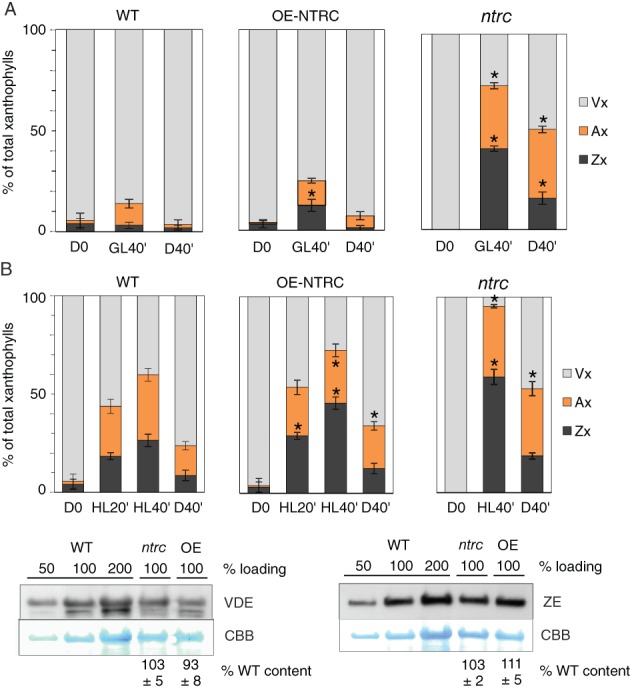
Accumulation of xanthophyll pigments in NTRC‐transgenic plants. (A, B) Relative accumulation of xanthophylls in growth (A) and high light conditions (B) and after transition of plants to darkness. Violaxanthin (Vx), antheraxanthin (Ax) and zeaxanthin (Zx) contents were determined with HPLC after 8 h of darkness (D0), after 40 min in growth light (GL40'), 20 (HL20') and 40 min in high light (HL40', 600 μmol photons m^−2^ s^−1^), and after subsequently transfer of plants back to darkness for 40 min (D40'). The data is shown as relative to total xanthophyll content (Vx + Ax + Zx = 100%). Values are averages from three to seven biological replicates ± SE, and statistically significant differences to WT according to Student's *t*‐tests (*P* < 0.05) are marked with *. (C) Protein content of VDE and ZE in WT, *ntrc* and OE‐NTRC. Representative immunoblots (loaded on base of protein content), CBB staining used as loading control, and quantified averages ± SE from of three (ZE) and four (VDE) replicates as percentage from WT are shown.

Lumenal acidification induces qE both via the PsbS protein (Li et al. 2000), as well as through activation of the xanthophyll cycle enzyme VDE, which converts Vx to Zx (Jahns and Holzwarth 2012). Interestingly, PsbS content was increased in *ntrc* while no difference in PsbS content was detected between OE‐NTRC and WT (Fig. 1E). Since both VDE and ZE are redox‐regulated (Hall et al. 2010, Simionato et al. 2015, Da et al. 2018), NTRC may influence the induction of qE through altered regulation of the xanthophyll cycle. To investigate this possibility, we measured the content of Vx, Ax and Zx in *ntrc* and OE‐NTRC leaves. The *ntrc* mutant accumulated significantly higher levels of both Ax and Zx than WT during 40 min in GL or HL and both Ax and Zx remained elevated in *ntrc* even 40 min after cessation of illumination (Fig. 2A,B), in line with the high NPQ observed. High accumulation of Zx was measured in GL and HL also in OE‐NTRC (Fig. 2A,B). No differences to WT were detected in the amount of VDE or ZE enzymes in *ntrc* or OE‐NTRC (Fig. 2C).

These results indicated that higher acidification of lumen in LL and GL in *ntrc* and in GL and HL in OE‐NTRC (Fig. 1D) increases the activity of VDE and induces higher accumulation of Zx in comparison to WT (Fig. 2A,B). In *ntrc*, the high accumulation of Zx in light together with higher PsbS content (Fig. 1E) likely contributes to the strong NPQ observed in all light intensities (Fig. 1; Naranjo et al. 2016). In contrast, ΔpH and relative Zx content were elevated in GL and HL in OE‐NTRC (Figs 1B and 2A,B), but NPQ was lower in comparison to WT (Fig. 1C).

### NTRC enhances phosphorylation‐dependent redistribution of excitation energy between the photosystems

The distribution of excitation energy between PSI and PSII by state transitions is another regulatory process of photosynthesis that has been proposed to be controlled by stromal thiol redox state (Rintamäki et al. 2000, Lemeille et al. 2009). Overexpression of NTRC causes elevation of chlororespiration, which results in reduction of the PQ pool and phosphorylation of LHCII proteins in darkness (Nikkanen et al. 2018). Therefore, we investigated whether the differential phosphorylation of LHCII proteins affects the relative size of the PSI antenna cross section or the capability of NTRC‐transgenic lines to perform state transitions.

**Figure 3:**
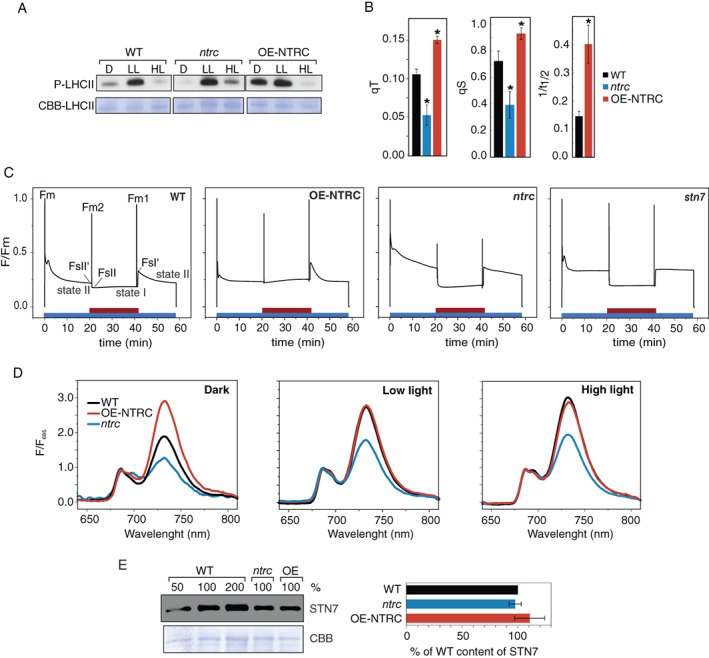
LHCII protein phosphorylation and state transitions in WT, *ntrc* and OE‐NTRC. (A) Determination of phosphorylation status of LHCII proteins in WT, *ntrc*, and OE‐NTRC. Thylakoid membranes were isolated after incubation for 2 h in darkness (D), low light (LL, 40 μmol photons m^−2^ s^−1^), and high light (HL, 600 μmol photons m^−2^ s^−1^). Thylakoid extracts with 0.4 μg of Chl were separated with SDS‐PAGE and phosphorylated proteins were detected with a Phosphothreonine‐specific antibody. Coomassie brilliant blue staining of LHCII on the membrane (CBB‐LHCII) was used as loading control. (B) The state transition parameters qT, qS and 1/*t*_1/2_ in WT, *ntrc* and OE‐NTRC. Parameters were calculated from Chl *a* fluorescence changes shown in (C) as follows: qT = (Fm1−Fm2)/Fm2) (Bennett 1979, Jensen et al. 2000), qS = (FsI′‐FsII′)/(FsI′−FsII) (Ruban and Johnson 2009), 1/*t*_1/2_ = inverse of the half time of fluorescence decay from FsI' to FsII level. The values are averages of measurements from five to six individual leaves ± SE. * indicates statistically significant difference to WT according to Student's *t*‐tests (*P* < 0.05). The 1/*t*_1/2_ parameter could not be quantified from *ntrc*, because fluorescence decay was too slow to reliably determine a half time. (C) Representative curves of Chl *a* fluorescence traces from WT, OE‐NTRC, *ntrc* and *stn7*. Red bar = far red light. (D) Chl *a* fluorescence emission spectra at 77 K from thylakoid membranes isolated after incubation for 2 h in darkness, LL, and HL. Excitation wavelength was 440 nm, and the spectra were normalized to the level of fluorescence at 685 nm. Averaged curves from three measurements are shown. (E) Protein content of STN7 in thylakoid extracts from WT, *ntrc* and OE‐NTRC. A representative immunoblot loaded on basis of protein content and quantified averages from five experiments ± SE are shown. Coomassie brilliant blue (CBB) staining was used as loading control.

**Figure 4:**
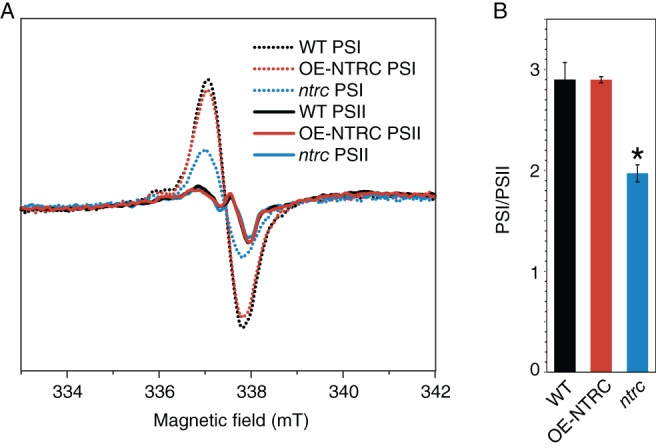
Determination of the ratio of functional PSI/PSII in WT, OE‐NTRC and *ntrc* thylakoids by EPR spectroscopy. (A) P700^+^ (PSI) and tyrosine D* (PSII) EPR spectra from isolated thylakoids of WT, OE‐NTRC and *ntrc*. The spectra are averages from three biological replicates and are normalized according to Chl *a* concentration. (B) Functional PSI/PSII ratios in WT, OE‐NTRC and *ntrc* thylakoids ± SE, calculated from (A). *indicates statistically significant difference to WT according to Student's *t*‐test (*P* < 0.05).

In agreement with the dark‐phosphorylation of LHCII proteins (Fig. 3A), measurement of Chl fluorescence at 77 K confirmed that the relative size of PSI antenna cross‐section was indeed increased in dark‐adapted OE‐NTRC leaves when compared to WT, while no difference was detected between illuminated WT and OE‐NTRC leaves (Fig. 3D). The size of the PSI antenna was lower both in dark‐adapted and illuminated *ntrc* leaves in comparison to WT (Fig. 3D). These results are in line with the significantly lower ratio of functional PSI/PSII complexes in *ntrc* than in WT or OE‐NTRC thylakoids as determined by EPR spectroscopy (Fig. 4), as well as with lowered PSI content at protein level in *ntrc* (Thormählen et al. 2015, Nikkanen et al. 2018).

We calculated the parameters related to state transitions from Chl *a* fluorescence, describing the extent of decrease in the size of PSII antenna cross section (qT), effectiveness of state transitions (qS), and the rate of steady‐state fluorescence change (1/*t*_1/2_) (Bennett 1979, Jensen et al. 2000, Ruban and Johnson 2009). Our results show that OE‐NTRC has a significantly enhanced, and *ntrc* has a significantly reduced, capability to perform state transitions when compared to WT (Fig. 3B,C and S1). As expected, the *stn7* mutant was unable to perform state transitions. Both the effectiveness (qS parameter) and the rapidness of state transitions (1−*t*_1/2_ parameter) were enhanced in OE‐NTRC (Fig. 3B). No changes in STN7 content were detected in OE‐NTRC or *ntrc* (Fig. 3E).

### Oxidation of PSI by far red light is accelerated by NTRC overexpression

To further investigate how NTRC affects the redox poise between the two photosystems and the stroma, we measured the FR‐induced redox changes of P700, PC and Fd. The redox states of PC, P700 and Fd depend on the activity of acceptor side reactions and on the processes delivering electrons to PSI, namely linear electron transfer from PSII and CEF. We used the Dual/Klas‐NIR spectrophotometer, which allows deconvolution of the PC, P700 and Fd signals from four absorbance difference changes at near‐infrared wavelengths (Klughammer and Schreiber 2016, Schreiber 2017).

**Figure 5:**
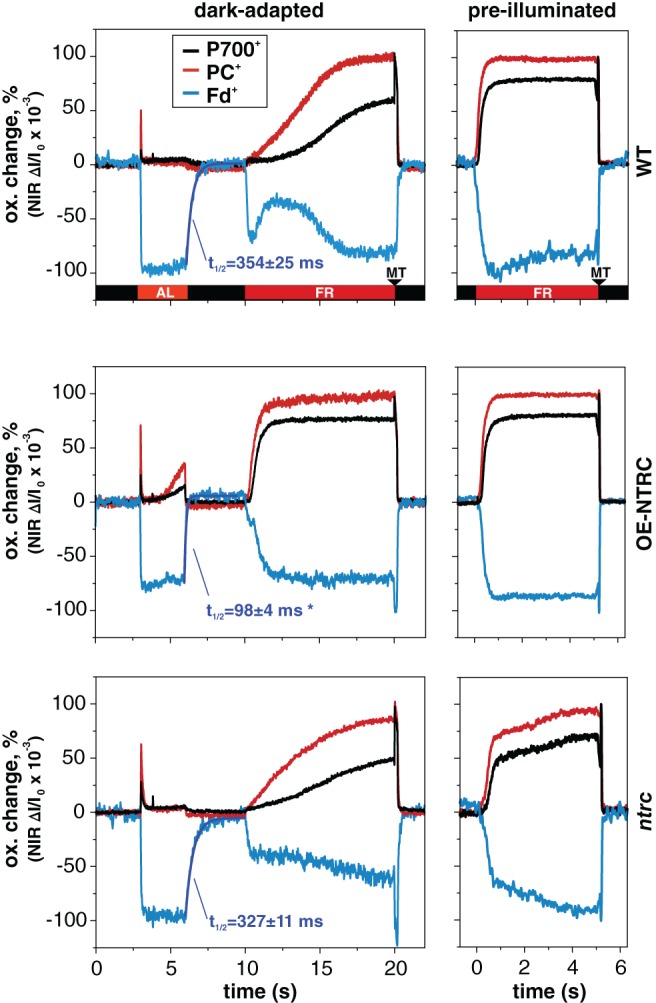
Oxidation and reduction kinetics of P700 (black), PC (red) and Fd (cyan) upon FR illumination in dark‐adapted leaves and leaves pre‐illuminated with 22 μmol photons m^−2^ s^−1^ for 10 min. Prior to FR‐illumination, the dark‐adapted leaves were first illuminated with 200 μmol photons m^−2^ s^−1^ of actinic light (AL) for 3 s to induce maximal reduction of Fd. A multiple‐turnover flash (MT) was administered after FR to induce maximal oxidation of P700 and PC. Representative curves normalized to maximal oxidation of P700 and PC and maximal reduction of Fd from measurements from six to 12 individual leaves are presented. Blue curves are first‐order exponential fits to post‐AL re‐oxidation kinetics of Fd, and average half times of Fd re‐oxidation (*t*_1/2_ ± SE) calculated from measurements from six to 12 individual leaves are shown. Statistically significant difference to WT according to Student's *t*‐test (*P* < 0.05) is indicated by *.

FR‐induced reduction of Fd and oxidation of PC and P700 in dark‐adapted leaves was affected by absence or overexpression of NTRC (Fig. 5). The initial reduction of Fd during AL and FR illumination of dark‐adapted WT was fast and likely caused by acceptor side limitation due to inactive Calvin‐Benson cycle.. The transient re‐oxidation of Fd in FR‐illuminated WT has been shown to disappear in anoxic conditions, suggesting that it may be caused by reduction of oxygen via the water–water cycle (Schreiber 2017). In dark‐adapted OE‐NTRC leaves, the re‐oxidation of Fd was faster than in WT in darkness after short AL illumination (half‐time of Fd re‐oxidation was 354 ± 25 in WT and 98 ± 4 ms in OE‐NTRC, Fig. 5). Under FR illumination, oxidation of PC and P700 was accelerated in comparison to WT, while Fd was reduced more slowly than in WT upon onset of FR illumination (Fig. 5). These kinetic changes were likely due to the higher activity of reactions on the acceptor side of Fd, namely the enzymes in Calvin‐Benson cycle (Nikkanen et al. 2016) as well as NDH‐dependent CEF (Nikkanen et al. 2018), and increased size of PSI antenna cross‐section (Fig. 3D). PC and P700 oxidations during FR were slower in dark‐adapted *ntrc* than in WT (Fig. 5).

We also compared the FR‐induced redox changes of P700, PC and Fd in leaves pre‐illuminated at LL. The redox kinetics were remarkably similar in both dark‐adapted and pre‐illuminated OE‐NTRC leaves (Fig. 5), while clearly slower kinetics were observed in dark‐adapted than pre‐illuminated WT leaves. This further supports the conclusion that the differences in kinetics observed between dark‐adapted WT and OE‐NTRC leaves resulted from dark‐activation of processes on the acceptor side of Fd in OE‐NTRC.

## Discussion

Recent reports on chloroplast TRX systems have emphasized their crucial role in the regulation of processes that allow plants to cope with changes in light conditions (Carrillo et al. 2016, Nikkanen et al. 2016, Thormählen et al. 2017, Da et al. 2018, Nikkanen et al. 2018). These include regulatory and photoprotective processes of photosynthetic electron transfer, ATP synthesis as well as carbon fixation and secondary carbon metabolism (for reviews, see Michelet et al. 2013, Nikkanen and Rintamäki 2014, Geigenberger et al. 2017). Accordingly, modifications of TRX activity alter the redox poise between the photosynthetic electron transfer chain and stromal electron sinks (Rey et al. 2013, Thormählen et al. 2015, Carrillo et al. 2016, Nikkanen et al. 2016, Thormählen et al. 2017, Nikkanen et al. 2018).

In the current study, we have investigated the molecular background of altered induction, relaxation and magnitude of NPQ in NTRC‐transgenic lines under various light intensities. Our results indicate that both in *ntrc* and OE‐NTRC correlation between the generation of trans‐thylakoid *pmf* and the accumulation of xanthophylls with the induction of NPQ is partially released (Figs 1 and 2). Our data points to the existence of a ΔpH‐independent inhibitory mechanism of NPQ that is less active in the *ntrc* mutant but is likely constitutively active in the OE‐NTRC line. We also demonstrate that the modified NTRC content alters the capacity to perform state transitions in thylakoid membranes. NTRC overexpression significantly enhanced, while NTRC knockout impaired the plant's ability to redistribute excitation energy between PSII and PSI (Fig. 3).

### Stromal thiol redox state controls NPQ through multiple redox‐dependent mechanisms

The *ntrc* knockout line suffers from excessive NPQ especially under LL (Fig. 1), while lower steady‐state level of NPQ was observed in OE‐NTRC in HL (Fig. 1; Nikkanen et al. 2016). Translocation of protons by linear and cyclic electron transfer is coupled to generation of trans‐thylakoid *pmf*, which induces the qE component of NPQ via protonation of PsbS and accumulation of Zx in thylakoid membranes (Jahns and Holzwarth 2012, Niyogi and Truong 2013). In OE‐NTRC, an elevated *pmf* correlates with the rise of NPQ during the first minute of photosynthetic induction (Fig. 1B; Nikkanen et al. 2018), but at steady‐state NPQ is similar to WT in GL and lower than WT in HL despite higher generation of *pmf* and higher accumulation of Zx (Figs 1 and 2). High accumulation of de‐epoxidated xanthophyll pigments (Ax and Zx) in OE‐NTRC in comparison to WT implies an elevated activity of the VDE enzyme, likely due to the stronger acidification of lumen (Fig. 1D). Alternatively, the activity of ZE enzyme on the stromal side of thylakoids may be lower in OE‐NTRC. Reduction of ZE by NTRC has been demonstrated in vitro (Naranjo et al. 2016), and may induce partial inhibition of the enzyme comparable to HL‐induced inactivation of ZE reported previously (Reinhold et al. 2008). In summary, as the higher steady‐state ΔpH and content of Zx in OE‐NTRC did not induce higher NPQ than in WT (Figs 1 and 2), our results suggest active downregulation of NPQ via a ΔpH‐independent mechanism that is linked to stromal thiol redox state in OE‐NTRC.

In the highly pleiotropic *ntrc* mutant line, higher NPQ may be caused by changes in multiple factors (see discussion in Nikkanen et al. 2018). The large increase in NPQ in LL is caused by the acidification of lumen (Fig. 1) due to impaired reduction of CF_1_γ and consequent low activity of the ATP synthase (Carrillo et al. 2016, Nikkanen et al. 2016), increased CEF due to accumulation of reduced Fd and H_2_O_2_ (Nikkanen et al. 2018), as well as low activity of the Calvin‐Benson cycle (Nikkanen et al. 2016, Pérez‐Ruiz et al. 2017). In GL and HL, lumenal acidification, however, does not fully explain the NPQ values in *ntrc*, since ΔpH is similar to WT in these light conditions (Fig. 1D). The high NPQ and its slow relaxation in darkness in *ntrc* may also be due to higher accumulation of de‐epoxidated xanthophylls both in GL and HL (Fig. 2A,B). Elevated content of PsbS (Fig. 1E) likely partly explains the high NPQ, as elevated PsbS amount has been shown to result in higher NPQ (Li et al. 2002). As *ntrc* has both a diminished PSI/PSII ratio (Figs 3D and 4) and PSI protein content (Thormählen et al. 2015, Nikkanen et al. 2018), expression of *PSBS* may be upregulated in order to protect PSI from photodamage by enhanced NPQ. Accordingly, in *ntrc psbs* double mutants the excessive induction of NPQ as well as the impairment of growth are partially (but not completely) alleviated in comparison to the *ntrc* single mutant (Naranjo et al. 2016).

Based on our results from OE‐NTRC and *ntrc* together with other recent reports on *ntrc* mutant, we suggest that stromal thiol redox state regulates the induction of NPQ via multiple mechanisms. Firstly by affecting *pmf* generation through control of the ATP synthase and CEF pathways (Carrillo et al. 2016, Naranjo et al. 2016, Nikkanen et al. 2016, Nikkanen et al. 2018), secondly through regulation of the xanthophyll cycle (Da et al. 2018), and thirdly, through a new type of ΔpH‐independent PSII antenna quenching mechanism involving lumenal lipocalin and named qH (Malnoë et al. 2017). Suppression of qH occurs via a thioredoxin‐like protein SOQ1 in thylakoid membranes (Brooks et al. 2013). Mutants lacking SOQ1 exhibit an elevated, slowly reversible form of NPQ that is independent of ΔpH or zeaxanthin formation and persists even in the absence of the PsbS protein in *soq1 npq4* double mutants (Brooks et al. 2013, Malnoë et al. 2017). The kinetics of NPQ relaxation in *soq1* (Brooks et al. 2013) resemble those in *ntrc* (Fig. 1B). As SOQ1 was identified as a potential NTRC interactor in our Co‐IP/MS screening (Nikkanen et al. 2018), it is possible that the activation of this mechanism is impaired in *ntrc*, contributing to the high NPQ observed (Fig. 1). Conversely, in OE‐NTRC the SOQ1 system may be hyperactive, resulting in lower NPQ than expected based on the magnitude of the *pmf* (Fig. 1) and the content of de‐epoxidated xanthophylls (Fig. 2). It is unlikely that NTRC would directly control the function of SOQ1, because the TRX‐like domain resides in the lumen (Brooks et al. 2013). Instead, NTRC likely affects the amount of reducing equivalents transported from stroma to the lumen via the CcdA–HCF164 pathway (Motohashi and Hisabori 2006, Motohashi and Hisabori 2010).

### NTRC overexpression enhances PSI oxidation and the ability to re‐distribute excitation energy between PSII and PSI

In WT plants, the function of light‐dependent redox regulation of thylakoid electron transfer and stromal carbon metabolism is to enable efficient photoprotection and physiological plasticity in naturally fluctuating light conditions (recently reviewed by Geigenberger et al. 2017). In more stable laboratory conditions or controlled agricultural environments, strict regulation is often redundant and actually limits plant growth (Kromdijk et al. 2016). Overexpression of NTRC overrides the natural light‐dependency of thiol regulation of these processes by elevating the activity of chloroplast TRX systems regardless of light conditions (Nikkanen et al. 2016, Nikkanen et al. 2018). This is reflected in the finding that in dark‐adapted OE‐NTRC leaves, electrons were rapidly transferred from PSI to stromal acceptors upon illumination (Fig. 5). This modification of the redox kinetics of PSI is likely due to activation of processes on the acceptor side of Fd in dark‐adapted OE‐NTRC, namely the enzymes in Calvin‐Benson cycle (Nikkanen et al. 2016) as well as NDH‐dependent CEF (Nikkanen et al. 2018). Moreover, LHCII proteins were phosphorylated in dark‐adapted OE‐NTRC leaves (Fig. 3A), which likely caused the increase in the relative size of the antenna cross‐section of PSI (Fig. 3D), and contributed to a rapid oxidation of PSI components upon exposure to FR light (Fig. 5). In contrast, a decreased ratio of functional PSI/PSII (Fig. 4) and increased acceptor side limitation in *ntrc* (Nikkanen et al. 2016) resulted in slower oxidation of PC and P700 by FR illumination (Fig. 5).

Our experiments indicate that chloroplast thiol redox state also modifies the capacity of state transitions (Fig. 3). OE‐NTRC had an enhanced and *ntrc* an impaired capacity, effectiveness and rapidness of state transitions (Fig. 3B,C). These results show that chloroplast thiol‐redox state has either a direct or indirect effect on the ability to re‐distribute excitation energy among PSII and PSI. In *ntrc*, this is likely at least partially caused by lowered ratio of functional PSI/PSII (Fig. 4). Accordingly, impaired state‐transitions may partly explain the higher sensitivity of *ntrc* to fluctuating light conditions (Thormählen et al. 2017). In OE‐NTRC, the ratio of functional PSI/PSII does not differ from WT (Fig. 4), but detachment of LHCII trimers from PSII core and/or their association with PSI likely occurs more readily than in WT.

The impact of NTRC content on the ability to control the distribution of excitation energy between PSII and PSI is unlikely to derive from altered activity of the LHCII kinase STN7 (Bellafiore et al. 2005), because phosphorylation of LHCII proteins in light did not differ from WT in OE‐NTRC or *ntrc* (Fig. 3A). Moreover, while the phosphorylation of LHCII proteins has been shown to be thiol‐sensitive (Rintamäki et al. 2000, Lemeille et al. 2009), no light‐dependent changes in STN7 thiol redox state have been observed (Shapiguzov et al. 2016).

Stromal thiol redox state may also control state transitions by STN7‐independent mechanisms. For instance, the monomeric LHC protein CP29.3, one of the three CP29 isoforms, was identified recently in our Co‐IP/MS screening as a potential NTRC interactor (Nikkanen et al. 2018). Triple knockout mutants of all three CP29 isoforms show, similarly to OE‐NTRC, faster kinetics of state transitions as well as decreased NPQ and increased P700 oxidation (de Bianchi et al. 2011). CP29 has been suggested to be an interaction partner with PsbS (Teardo et al. 2007) and it is part of a hetero‐oligomeric complex of PSII antenna, whose dissociation is essential for induction of qE (Betterle et al. 2009). Interestingly, CP29 contains a single conserved cysteine residue, which has been identified as a target of endogenous S‐nitrosylation (Puyaubert et al. 2014). Hypothetical redox‐dependency of CP29 function could therefore contribute to explain the state transition and NPQ phenotypes of NTRC‐mutated plants.

These observations demonstrate that the electron sink capacity of the stroma is highly dependent on TRX activity, and that TRXs provide a buffering system that allows maintenance of photosynthetic redox balance during natural fluctuations in light conditions. Conversely, overriding of these mechanisms by NTRC overexpression likely contributes to the enhanced growth of OE‐NTRC plants at least in laboratory conditions (Toivola et al. 2013).

## Author contributions

L.N. and E.R. designed the experiments. L.N., M.G., J.T. and A.T. performed the experiments. L.N., M.G., J.T., A.T. and E.R. analyzed the data. L.N. and E.R. wrote the article.

## Supporting information


**Fig. S1.** Representative chlorophyll a fluorescence traces from experiments to determine state transition capacities of WT, *ntrc* and OE‐NTRC leaves.
