# Supplementary material for: Multilevel regulation of non‐photochemical quenching and state transitions by chloroplast NADPH‐dependent thioredoxin reductase
Source: Physiol Plant. 2019 Feb 8;166(1):211–25. doi: 10.1111/ppl.12914 (PMC6850073; doi:10.1111/ppl.12914)
Supplement: Supplementary file 1 — Fig. S1. Representative chlorophyll a fluorescence traces from experiments to determine state transition capacities of WT, ntrc and OE‐NTRC leaves. [file PPL-166-211-s001.pdf]

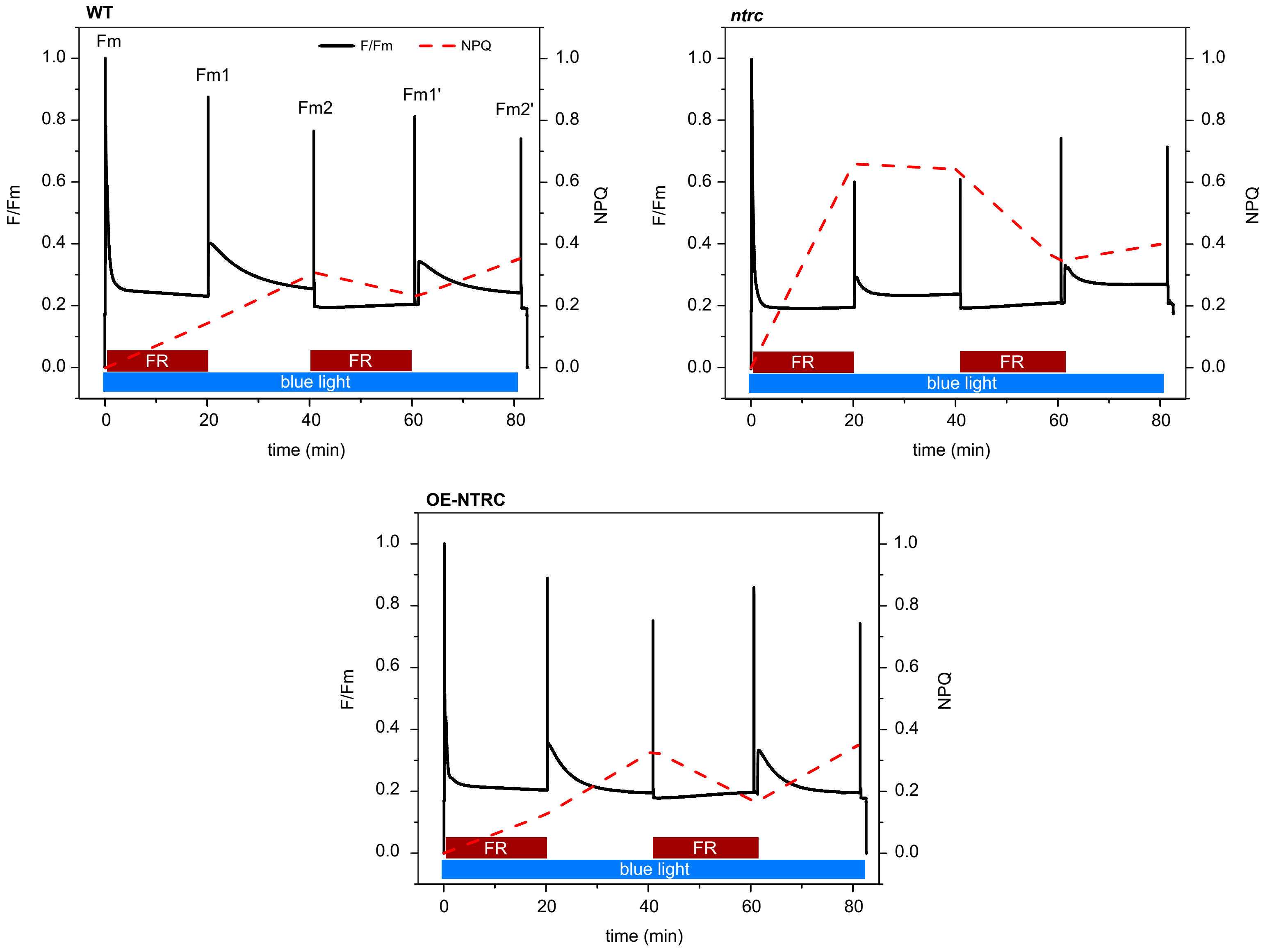

Supplemental Figure S1. Representative chlorophyll a fluorescence traces from experiments to determine state transition capacities of WT, *ntrc* and OE-NTRC leaves.

The *ntrc* mutant suffered from high NPQ in the low intensity of blue actinic light used in the experiment in Fig. 3C. In order to reliably determine qT in *ntrc*, this experiment shown here was performed as in Fig. 3C, except that an additional 20 min illumination period with blue light (35 µmol photons m^{-2} s^{-1}) supplemented with far red light (FR) was applied after the initial saturating pulse. The qT parameter was calculated from the fluorescence difference between Fm2 and Fm1' (qT=(Fm1' – Fm2) / Fm2). The values of qT that were obtained from WT and OE-NTRC leaves were similar to the values obtained from the shorter experiment shown in Fig. 3C. Dotted lines depict the value of NPQ at the timepoints of saturating pulses. For each line, three individual leaves were measured.
